# Assessment of prevalence and risk factors of diabetes and pre-diabetes in South Africa

**DOI:** 10.1186/s41043-022-00281-2

**Published:** 2022-03-02

**Authors:** Nina Grundlingh, Temesgen T. Zewotir, Danielle J. Roberts, Samuel Manda

**Affiliations:** 1 School of Mathematics, Computer Science and Statistics, University of KwaZulu-Natal, University Road, Westville, Private Bag X54001, Durban, 4000 South Africa; 2 Biostatistics Research Unit, South African Research Medical Council, 1 Soutpansberg Road, Private Bag x385, Pretoria, 0001 South Africa; 3 Present Address: School of Data Science and Analytics, Kennesaw State University, Kennesaw, Georgia 30144 USA; 4 Department of Statistics, University of Pretoria, Pretoria, South Africa

**Keywords:** Generalized additive mixed models, Spatial autocorrelation, Survey logistic regression

## Abstract

**Background:**

Diabetes prevalence, as well as that of pre-diabetes, is rapidly increasing in South Africa. Individuals with pre-diabetes have a high risk of developing type 2 diabetes, which is reversible with a change in lifestyle. If left untreated, diabetes can lead to serious health complications. Our objective was to assess the prevalence of diabetes and pre-diabetes, and to investigate the associated risk factors of each in the South African population.

**Method:**

This study made use of the South African Demographic Health Survey 2016 data. The study participants included 6442 individuals aged 15 years and older. A generalized additive mixed model was employed to account for the complex survey design of the study as well as well spatial autocorrelation in the data.

**Results:**

The observed prevalence of pre-diabetes and diabetes was 67% and 22%, respectively. Among those who had never been tested for diabetes prior to the survey, 10% of females and 6% of males were found to be diabetic, and 67% of both males and females were found to be pre-diabetic. Thus, a large proportion of the South African population remains undiagnosed. The model revealed both common and uncommon factors significantly associated with pre-diabetes and diabetes. This highlights the importance of considering diabetic status as a three-level categorical outcome, rather than binary. In addition, significant interactions between some of the lifestyle factors, demographic factors and anthropometric measures were revealed, which indicates that the effects each these factors have on the likelihood of an individual being pre-diabetic or diabetic is confounded by other factors.

**Conclusion:**

The risk factors for diabetes and pre-diabetes are many and complicated. Individuals need to be aware of their diabetic status before health complications arise. It is therefore important for all stakeholders in government and the private sector of South Africa to get involved in providing education and creating awareness about diabetes. Regular testing of diabetes, as well as leading a healthy lifestyle, should be encouraged.

## Background

Diabetes mellitus (diabetes) is a metabolic disorder in which the body becomes resistant to the effect of insulin or does not produce enough of this hormone to process glucose [1]. As a consequence, there is a buildup of glucose, or sugar, in the body which can lead to serious health complications. The number of people with diabetes globally has risen from 108 million in 1980 to 463 million in 2019, which resulted in an increase in the prevalence in adults over the age of 18 from 4.7% in 1980 to 9.3% in 2019 [2].

Diabetes was the second leading underlying cause of death in South Africa in 2016 and 2017 [3]. Furthermore, it was found to be the number one leading underlying cause of death for females [3]. South Africa has seen a rapid increase in the prevalence of diabetes, where it has almost tripled from 4.5% in 2010 to 12.7% in 2019. It was estimated that of the 4.58 million people between 20 and 79 years old with diabetes in South Africa in 2019, 52.4% were undiagnosed [4]. According to the Indigo Wellness Index in 2019, South Africa was named the “unhealthiest country on earth”. This ranking was based on measures that included blood glucose (diabetes risk) and obesity, among others [5]. Accordingly, the South African government implemented a sugar tax in 2018, where sugar-sweetened beverages are now subjected to a tax based on their sugar content [6]. This was done in an effort to curb the overconsumption of sugar, which has been linked to the growing burden of non-communicable diseases, such as diabetes, in the South African population [6].

Two main types of diabetes exist: Type 1 diabetes mellitus (T1DM) and Type 2 diabetes mellitus (T2DM). T2DM is more common and is believed to account for over 90% of diabetes cases [7]. T1DM is due to an autoimmune disease where individuals have low insulin levels and thus cannot adequately regulate their blood glucose levels, whereas T2DM is due to insulin resistance, where the body does not use the insulin produced as well as it should therefore lead to high blood glucose levels [1]. Treatment for T1DM involves exogenous insulin and can never be reversed. T2DM treatment involves a change in lifestyle and, in particular, in diet. T2DM can be reversed [8]. However, if left untreated, diabetes (T1DM or T2DM) can lead to serious nerve and blood vessel damage which could result in physical repercussions to different parts of the body. Many studies have shown that the majority of individuals with diabetes, particularly T2DM, are prone to multiple comorbidities [9–11]. Furthermore, diabetes has been reported as one of the most common comorbidities in patients infected with COVID-19 and patients with diabetes are associated with an increased risk of death due to COVID-19 [12, 13]. However, it has been suggested that if a patient’s diabetes is well-managed, then the risk of experiencing severe complications from COVID-19 is about the same as the general population [14]. Thus, it is important for individuals to be aware of their diabetic status before health complications arise.

The health complications caused by diabetes has a serious effect on an individual and their family as it could keep them from work and therefore restrict their earnings. This loss of earnings can also negatively affect the country’s economy. In addition, the health implications can put a strain on the country’s resources. In 2015, it was estimated that the economic cost due to diabetes in sub-Saharan Africa was 1.2% of the gross domestic product, where these countries generally spend 5.5% of their gross domestic product on health in total [15].

Previous studies on diabetes have focused on specific groups which were not representative of South Africa as a whole [16–19]. Our study is based on the South African Demographic and Health Survey. This study aimed to assess the prevalence of diabetes and pre-diabetes and investigate the associated risk factors of each in the South African population.

## Methods

### Study area and data

South Africa is a country on the southernmost tip of the African continent and is comprised of nine provinces. The South African population is made up of individuals with a wide variety of cultures, languages, and religions. This study utilized data from the nationally representative South African Demographic and Health Survey (SADHS) which was carried out from 27 June 2016 to 4 November 2016. This survey was administered by Statistics South Africa, in partnership with the South African Medical Research Council, at the request of the National Department of Health. The primary objective of the survey was to provide up-to-date estimates of basic demographic and health indicators in South Africa.

The survey followed a stratified two-stage sampling design where each province was stratified into urban, farm and traditional areas, excluding the Western Cape province, which does not have traditional residential areas. At the first stage of sampling, the primary sampling units were selected with a probability proportional to their size, where primary sampling units containing more dwelling units had a higher chance of being selected. The second stage consisted of systematic sampling to select a fixed number of 20 dwelling units per primary sampling units/cluster. Thereafter, all selected dwelling units were asked the Household Questionnaire, the Woman’s Questionnaire and the Caregiver’s Questionnaire. In addition, the even numbered dwelling units were asked the Man’s Questionnaire and had their biomarkers collected if written consent was given. Both the Woman’s and Man’s Questionnaires included a module on adult health in which only one individual aged 15 years or older in the household answered. The adult health module included information on smoking, alcohol consumption, dietary habits, health care seeking behaviours, and self-reported prevalence of a variety of non-communicable diseases. The Biomarker Questionnaire recorded data on biomarkers such as anthropometry, anaemia testing, blood pressure measurements and glycated haemoglobin (HbA1c) testing. More information pertaining to the SADHS 2016 can be found in [20].

### HbA1c testing

The HbA1c test is a diagnostic test for diabetes and measures how well glucose has been controlled in the body over a relatively long period of 120 days, the lifespan of red blood cells. HbA1c, known as glycated haemoglobin or haemoglobin A1c, occurs when the oxygen-carrying protein in red blood cells (haemoglobin) becomes bonded with glucose in the bloodstream. This bonding is called glycation. The higher the blood glucose levels, the higher the number of glycated red blood cells which therefore results in a higher HbA1c level [21]. The HbA1c measure is simple and convenient as it does not require one to be fasting. An individual is classified as non-diabetic if his/her HbA1c<5.7%, pre-diabetic if his/her HbA1c is between and including 5.7-6.4% and diabetic if his/her HbA1c$\ge {6.5}\%$ as defined by the American Diabetes Association [22]. Pre-diabetes is the stage in which an individual’s blood sugar level is high but not high enough to be classified as diabetic. Such individuals are at an increased risk of future progression of full-fledged T2DM [8]. It should be noted that T1DM and T2DM cannot be distinguished in those who were classified as diabetic as per the HbA1c test. However, pre-diabetes can only develop into T2DM [23]. Thus, pre-diabetes should not be overlooked.

### Variables of interest

The outcome variable in this study was the diabetic status of persons aged 15 years and older categorized into three: non-diabetic, pre-diabetic and diabetic.

**Fig. 1:**
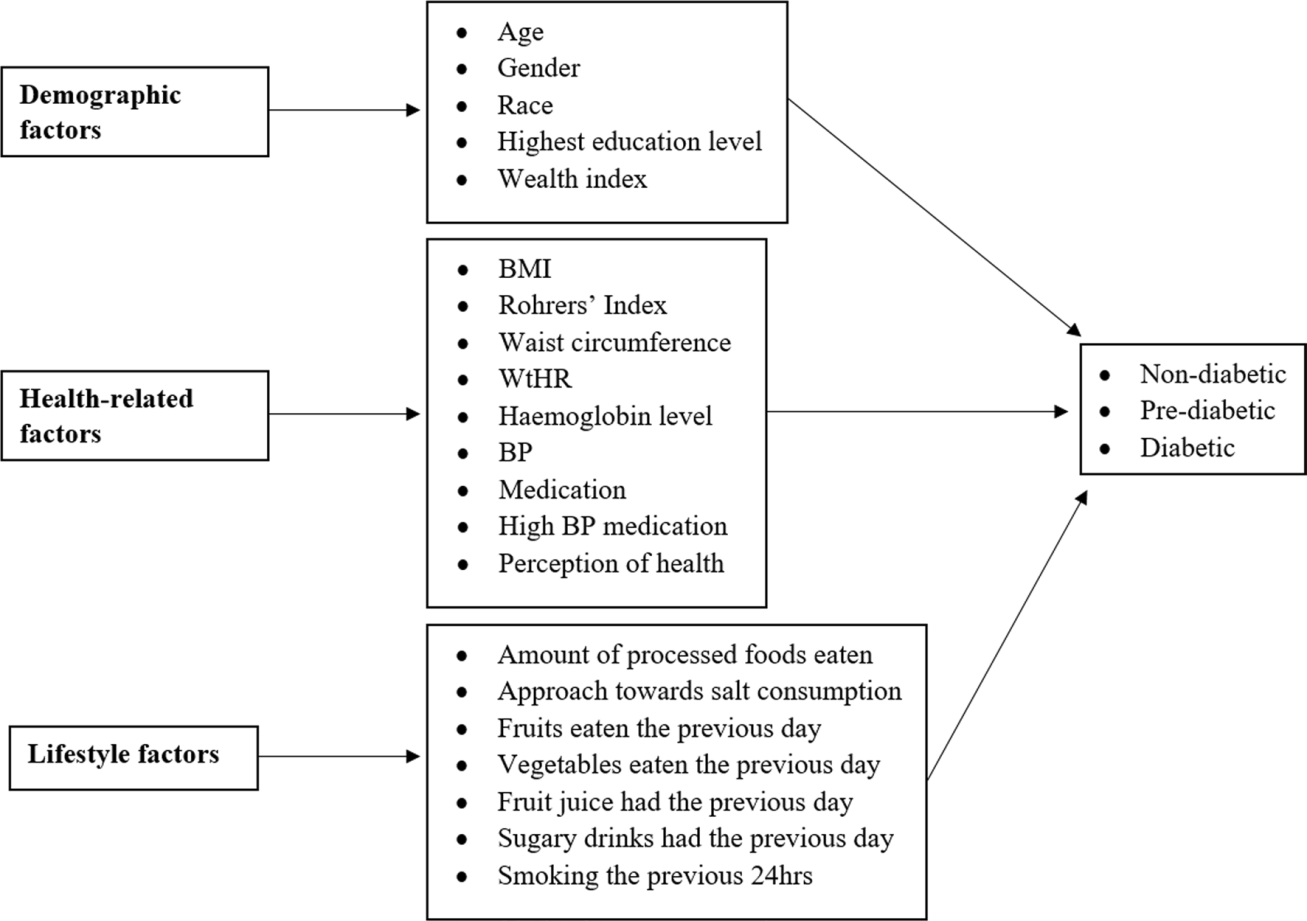
Conceptual framework of variables of interest for diabetes

The most important determinants of diabetes from various literature reviews [16, 17, 24, 25] were included as well as those variables that were expected to be determinants. Significant determinants of diabetes found in previous studies include race, age, central obesity, consumption of sugar and carbohydrates [16, 17, 26, 27]. The conceptual framework (Fig. 1) depicts the explanatory variables at individual and household levels that were used in the models. The wealth index used in this study is a continuous standardized Z-score for each household that was calculated based on the number and kind of consumer goods owned in the household. Body mass index (BMI) is an anthropometric measure that utilizes height and weight to determine body fat. Three categories of BMI were considered: underweight, normal and overweight to obese based on the World Health Organization standards [28]. Rohrer’s Index is a measure of leanness (or corpulence) of a person and is also known as the Corpulence Index. Waist-to-height ratio (WtHR) is another anthropometric measure that is used to determine an individual’s lifestyle risk and their weight in relation to their body build. Blood pressure (BP) is the average blood pressure of an individual categorized as either normal or abnormal where abnormal represents the case in which the diastolic or systolic readings were out of range. Perception of health was determined on an individual level and was based on whether the individual believed they had good or excellent health (regarded as positive) or had average or bad health (regarded as negative). The amount of processed food eaten was determined at a household level. This is a continuous variable that looked at the number of processed foods consumed out of four varieties (packed chips, fast food, fried food and processed meat). Fruit and vegetable consumption were kept separate as fruit has a higher sugar content. In addition, fruit juice was kept separate from fruit consumption as its content is more concentrated in sugar and calories. The juicing process causes a loss in vitamins and fibre [29]. Approach towards salt consumption was considered positive if individuals had or believed they should reduce their salt intake and negative if otherwise.

### Statistical methods

The SADHS 2016 data is of a complex survey design in which multistage sampling, clustering and unequal weighting were involved, where samples were not collected in proportion to the population. Accordingly, it is essential to incorporate the sample design in the data analysis to make statistically valid inferences for the population by means of avoiding biased estimates of parameters and incorrect variance estimates [30]. The ordinal nature of diabetic status requires the use of ordinal logistic regression. As such, an ordinal survey logistic regression, which accounts for the sampling design, was adopted. However, the proportional odds assumption of this model was violated.

We then made use of a multinomial generalized linear mixed model (GLMM) with a generalized logit link. The cluster in which a person resided was included as a random effect to account for possible correlations in the observations, as it would be expected that those in the same cluster would be more alike than those from different clusters. In addition, the sampling weights were incorporated into the analysis. After fitting the multinomial GLMM, there was concern for spatial autocorrelation in the residuals due to the nature of the design of the study. Strongly correlated residuals reduce the statistical power of inference, making a model untrustworthy. We explored various methods of accounting for spatial autocorrelation in the residuals. However, incorporating longitude and latitude, based on the geographical coordinates of the clusters, as fixed spline effects in the model was the only method that sufficiently accounted for this spatial autocorrelation in the residuals. We utilized B-spline basis functions for longitude and latitude separately, with all other effects considered as linear. The resulting model is now referred to as a generalized additive mixed model (GAMM).

The GAMM has the following form:

1 $$\begin{aligned} \eta |\gamma =\alpha +f_{1}(x_{1})+f_{2}(x_{2})+...+f_{p}(x_{p})+z_{1}\gamma _{1}+z_{2}\gamma _{2}+...+z_{q}\gamma _{q} \end{aligned}$$

where $\eta$ is the link function, $\alpha$ is the intercept term, $f_{1}(x_{1})+f_{2}(x_{2})+...+f_{p}(x_{p})$ are nonlinear or linear functions of the fixed effects, $z_{1},z_{2},...,z_{q}$ are the design covariates for the random effects and $\gamma _{1},\gamma _{2},...,\gamma _{q}$ are random effects that are normally distributed with mean ${\mathbf {0}}$ and variance ${\mathbf {D}}$ [31]. Laplace approximation was used for maximum likelihood estimation [32].

We made use of SAS version 9.4 and ArcGIS for the analysis.

## Results

### Characteristics of the study sample

**Fig. 2:**
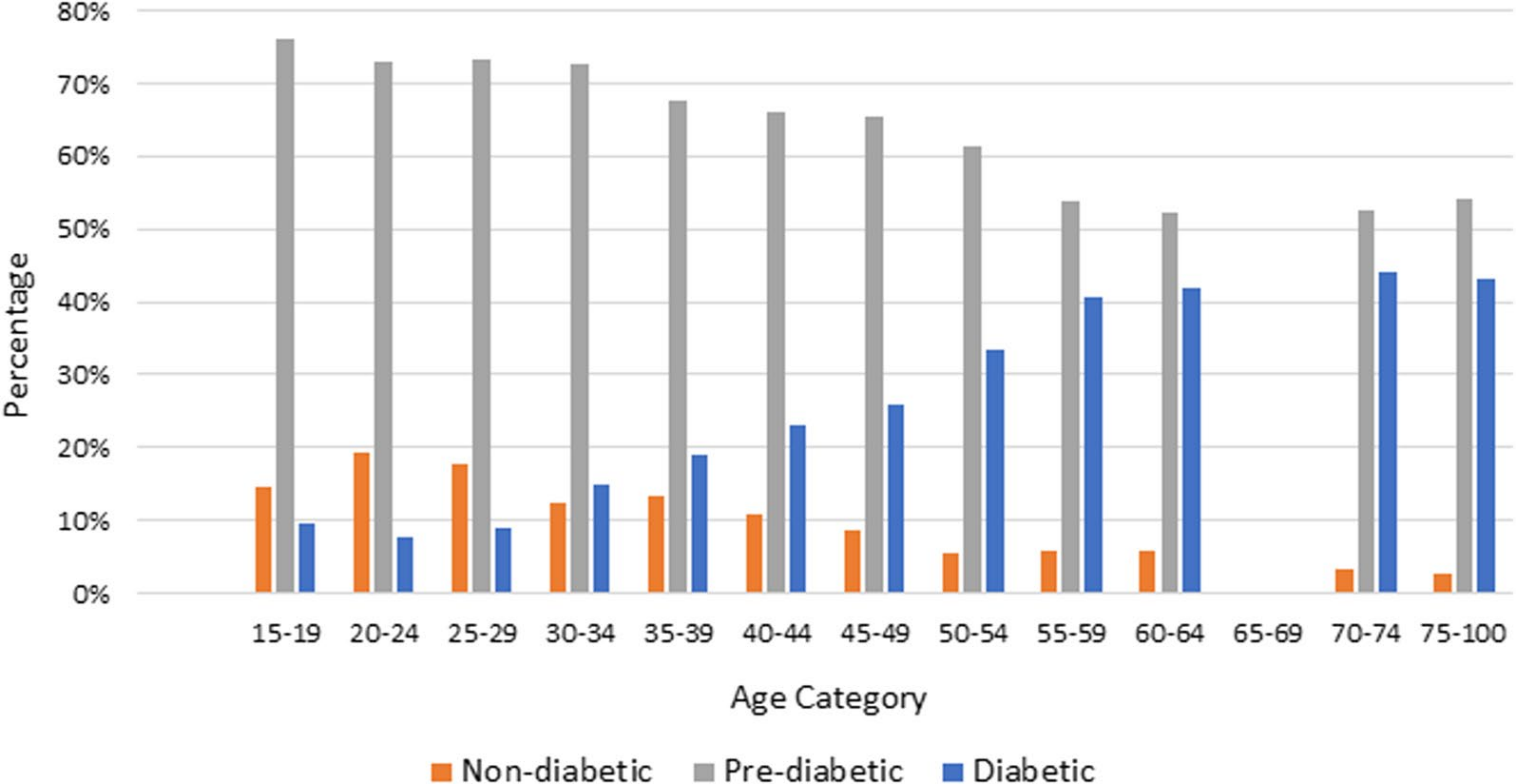
Diabetic status across different age groups

The final sample size in this study involved 3636 households made up of 6442 individuals that had consented to having their HbA1c tested and fully completed the questionnaires. From the sampled population, 11%, 67% and 22% were non-diabetic, pre-diabetic and diabetic, respectively. From which, 24.7% of females and 17.2% of males were found to be diabetic. Similarly, 64.9% of females and 69.5% of males were pre-diabetic. Figure 2 presents the frequency of non-diabetics, pre-diabetics and diabetics for each age group. A decreasing trend in non-diabetics and pre-diabetics is seen across the age groups; however, an increasing trend in diabetics is seen across the age groups. The SADHS questionnaires also included asking individuals whether or not they had ever been tested for diabetes prior to the survey. Among those who said no, 10% of females and 6% of males had a HbA1c result indicating they were diabetic, and 67% of both males and females had a HbA1c result indicating they were pre-diabetic [20].

**Table 1:** Summary of observed diabetes prevalence distribution by factor levels (N = 6442)

Factor	Level	Non-diabetic	Pre-diabetic	Diabetic	Total
Gender	Female	416 (10.4%)	2597 (64.9%)	989 (24.7%)	4002 (62.1%)
	Male	326 (13.4%)	1695 (69.5%)	419 (17.2%)	2440 (37.9%)
Race	Black/African	632 (11.1%)	3840 (67.3%)	1235 (21.6%)	5707 (88.6%)
	Other	110 (15%)	452 (61.5%)	173 (23.5%)	735 (11.4%)
Highest education level	Primary	528 (12.9%)	2824 (69.2%)	730 (17.9%)	4082 (63.4%)
	Secondary	155 (8.4%)	1126 (60.8%)	572 (30.9%)	1853 (28.8%)
	Other	59 (11.6%)	342 (67.5%)	106 (20.9%)	507 (7.9%)
Body mass index category	Underweight	79 (20.5%)	270 (69.9%)	37 (10.0%)	386 (6.0%)
	Normal	399 (15.1%)	1909 (72.0%)	342 (12.9%)	2650 (41.1%)
	Overweight to obese	264 (7.8%)	2113 (62.0%)	1029 (30.2%)	3406 (52.9%)
Blood pressure category	Normal	538 (13.2%)	2813 (69.2%)	714 (17.6%)	4065 (63.1%)
	Abnormal	204 (8.6%)	1479 (62.2%)	694 (29.2%)	2377 (36.9%)
Taking high blood pressure medication	No	672 (12.9%)	3634 (69.8%)	897 (17.2%)	5203 (80.8%)
	Yes	70 (5.6%)	658 (53.1%)	511 (41.2%)	1239 (19.2%)
Taking medication	No	661 (12.9%)	3549 (69.2%)	921 (17.9%)	5131 (79.6%)
	Yes	81 (6.2%)	743 (56.7%)	487 (37.1%)	1311 (20.4%)
Health perception	Poor	92 (9.9%)	586 (62.9%)	253 (27.2%)	931 (14.5%)
	Average	259 (10.9%)	1561 (65.9%)	548 (23.1%)	2368 (36.8%)
	Good	314 (12.7%)	1660 (67.3%)	493 (20.0%)	2467 (38.3%)
	Excellent	77 (11.4%)	485 (71.1%)	114 (16.9%)	676 (10.5%)
Ate fruit yesterday	Yes	318 (10.7%)	1962 (66.3%)	679 (22.9%)	2959 (45.9%)
	No	424 (12.3%)	2300 (66.6%)	729 (21.1%)	3453 (53.6%)
Ate vegetables yesterday	Yes	418 (11.0%)	2485 (65.6%)	885 (23.4%)	3788 (58.8%)
	No	324 (12.2%)	1807 (68.1%)	523 (19.7%)	2654 (41.2%)
Approach towards salt consumption	Positive	502 (11.1%)	2992 (66.1%)	1034 (22.8%)	4528 (70.3%)
	Negative	240 (12.5%)	1300 (67.9%)	374 (19.5%)	1914 (29.7%)
Had a sugary drink yesterday	Yes	247 (11.8%)	1408 (67.2%)	441 (21.0%)	2096 (32.5%)
	No	495 (11.4%)	2884 (66.4%)	967 (22.3%)	4346 (67.5%)
Had fruit juice yesterday	Yes	108 (13.3%)	515 (63.3%)	190 (23.4%)	813 (12.6%)
	No	634 (11.3%)	3777 (67.1%)	1218 (21.6%)	5629 (87.4%)
Smoked cigarettes the previous 24hrs	Yes	151 (15.4%)	686 (70.1%)	141 (14.4%)	978 (15.2%)
	No	591 (10.8%)	3606 (66.0%)	1267 (23.2%)	5464 (84.8%)

Table 1 shows the distribution of counts and the observed prevalence for each of the three diabetic statuses according to the categorical variables of interest. Of the individuals that have primary school as their highest level of education, 69.2% were pre-diabetic. From Table 1, it can be seen that a high percentage of individuals taking high blood pressure medication were diabetic (41.2%) as well as those taking any medication in general (37.1%). Of those individuals that believe to have an excellent perception of health, 71.1% were pre-diabetic. From our sampled population, 88.6% were Black/Africans of which 21.6% were diabetic and 67.3% were pre-diabetic. Of the individuals with a BMI classified as underweight to normal, 12.9% were diabetic and 71.8% were pre-diabetic. Similarly, of those with a BMI classified as overweight to severely obese, 30.2% were diabetic and 62.0% were pre-diabetic.

**Fig. 3:**
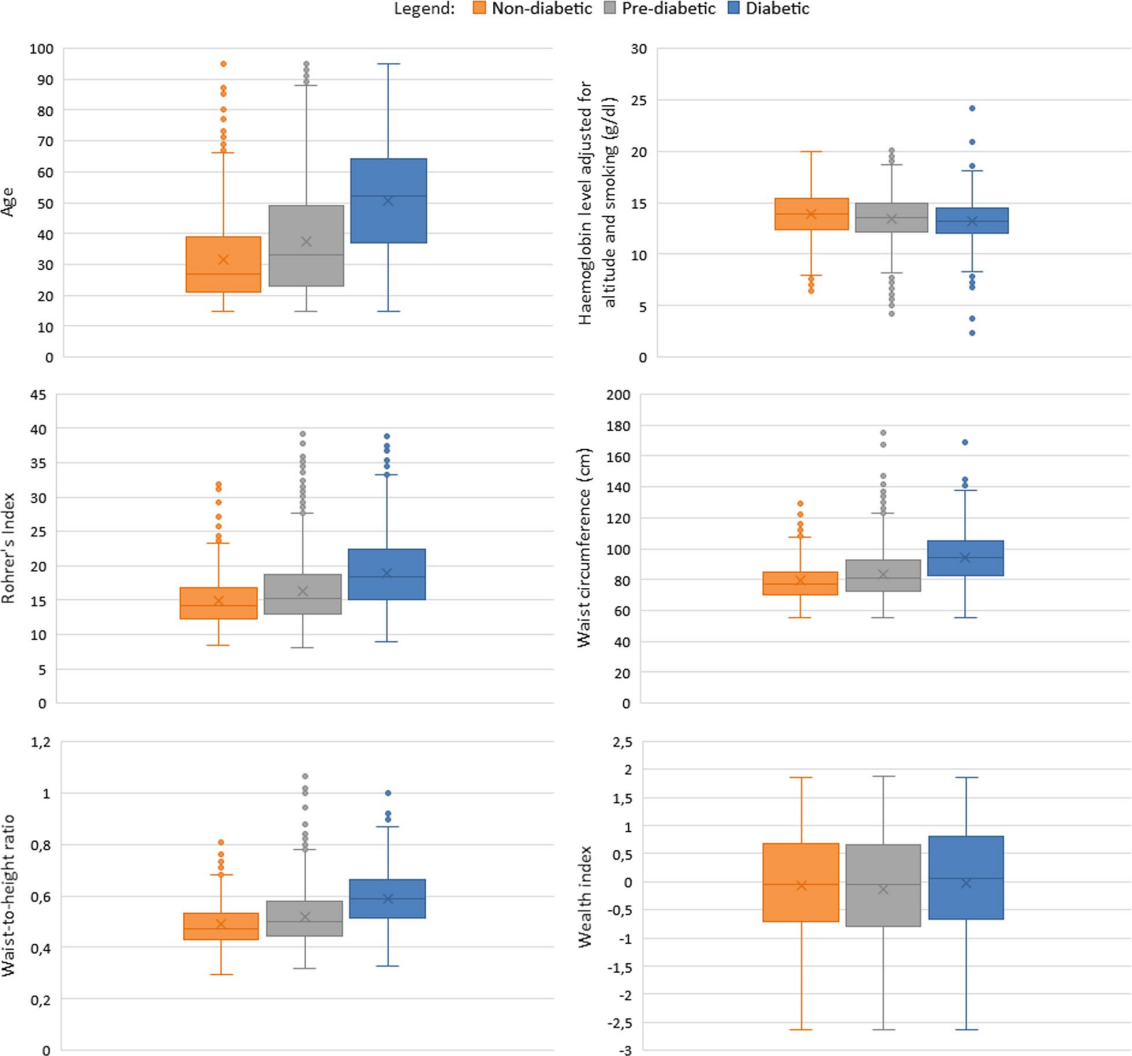
Boxplots for the continuous covariates by diabetic status

Figure 3 presents boxplots of the continuous covariates according to diabetic status. These boxplots display the minimum, first quartile, median, third quartile, maximum as well as the mean of the covariate according to diabetic status. In general, there is an increasing trend in age as a person’s diabetic status worsens. This was similarly seen with Rohrer’s index, waist circumference, and waist-to-height ratio. However, a slight decreasing trend is seen in a person’s haemoglobin level as their diabetic status worsens. Very little difference is seen in the wealth for the different diabetic statuses.

### GAMM applied to the SADHS 2016 data

All the explanatory variables of interest were incorporated in the multinomial GAMM with non-diabetic as the reference category. Before obtaining the results of the model estimates, we explored all two-way interaction effects between the explanatory variables in order to control for any confounding effects between them. The five significant interaction terms that yielded the lowest AIC were included in the final model. These included the interaction between WtHR and salt consumption, WtHR and BMI, consumption of salt and fruit juice, perception of health and fruit juice consumption, and education level and age.

#### Results of the main effects

Table 2 gives the estimated odds ratios and their 95% confidence intervals for variables not included in any interaction effect. Considering pre-diabetics vs non-diabetics, the factors significantly associated with pre-diabetes included gender, waist circumference, haemoglobin level (adjusted for altitude and smoking) and Rohrer’s index. Males were more likely to be pre-diabetic than non-diabetic (OR=1.326; 95% CI: 1.011-1.740) compared to females. For a unit increase in Rohrer’s index, individuals were more likely to be pre-diabetic rather than non-diabetic (OR=1.104; 95% CI:1.037-1.176). Similarly, for a unit increase in waist circumference, individuals were 1.041 times more likely to be pre-diabetic rather than non-diabetic (95% CI: 1.012-1.071). For a unit increase in haemoglobin level, individuals were 0.905 times less likely to be pre-diabetic rather than non-diabetic (95% CI: 0.860-0.952).

**Table 2:** Adjusted odds ratios (95% confidence intervals) for the Multinomial GAMM for variables not included in the interaction effects

Variable	Pre-diabetic	Diabetic
	OR (95% CI)	OR (95% CI)
*Gender (ref = Female)*		
Male	1.326 (1.011–1.740)*	1.439 (1.035–2.001)*
*Race (ref = Other)*		
Black/African	1.087 (0.743–1.590)	1.509 (0.953–2.389)
Rohrer’s Index	1.104 (1.037–1.176)*	1.058 (0.987–1.135)
Waist circumference	1.041 (1.012–1.071)*	1.048 (1.014–1.082)*
Haemoglobin level adjusted for altitude and smoking	0.905 (0.860–0.952)*	0.852 (0.802–0.905)*
*Blood pressure category (ref = Normal)*		
Abnormal	1.159 (0.934–1.439)	1.302 (1.014–1.671)*
*Taking high blood pressure medication (ref = No)*		
Yes	1.019 (0.731–1.420)	1.521 (1.054–2.196)*
*Taking Medication (ref =No)*		
Yes	1.294 (0.950–1.764)	1.487 (1.055–2.096)*
Household’s consumption of processed foods	1.088 (0.975–1.213)	1.009 (0.888–1.147)
*Household’s consumption of fruit the previous day (ref = Yes)*		
No	0.981 (0.814–1.182)	0.933 (0.743–1.170)
*Household’s consumption of vegetables the previous day (ref = No)*		
Yes	1.009 (0.837–1.218)	1.109 (0.882–1.394)
*Household’s consumption of sugary drinks the previous day (ref = No)*		
Yes	1.119 (0.921–1.358)	1.250 (0.988–1.583)
*Smoking the previous 24hrs (ref = No)*		
Yes	0.815 (0.634–1.045)	0.705 (0.510–0.974)*
Wealth index Z-score	0.932 (0.828–1.047)	1.010 (0.875–1.167)

Significant at 5% level of significance

Considering diabetics vs non-diabetics, the factors significantly associated with diabetes included gender, waist circumference, haemoglobin level (adjusted for altitude and smoking), blood pressure, taking high blood pressure medication, taking medication in general and smoking within the previous 24 hours of the survey. Males were more likely to be diabetic than non-diabetic (OR=1.439; 95% CI: 1.035-2.001) compared to females. Individuals taking high blood pressure medication were 1.521 times more likely to be diabetic than non-diabetic (95% CI: 1.054-2.196) compared to those not taking high blood pressure medication. Similarly, individuals taking any other medication were 1.487 times more likely to be diabetic than non-diabetic compared to those not taking medication (95% CI: 1.055-2.096). Individuals who had smoked the previous 24 hours were less likely to be diabetic rather than non-diabetic compared to those that had not smoked (OR=0.705; 95% CI:0.510-0.974).

#### Results of the interaction effects

**Fig. 4:**
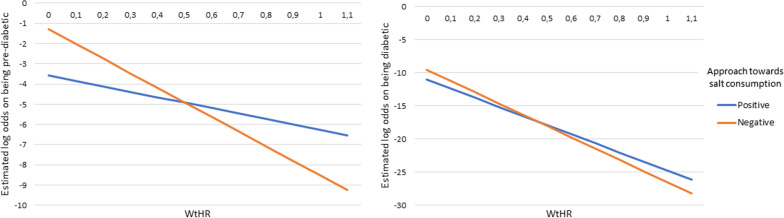
Interaction plot of waist-to-height ratio and salt consumption

**Fig. 5:**
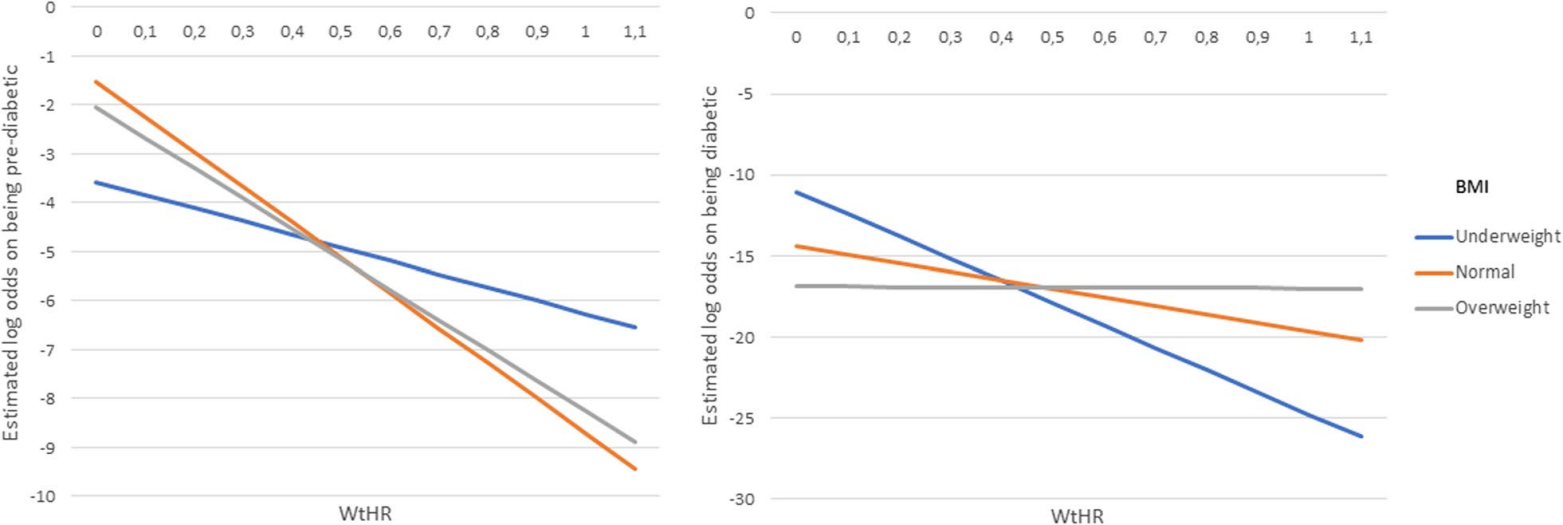
Interaction plot of waist-to-height ratio and body mass index category

**Fig. 6:**
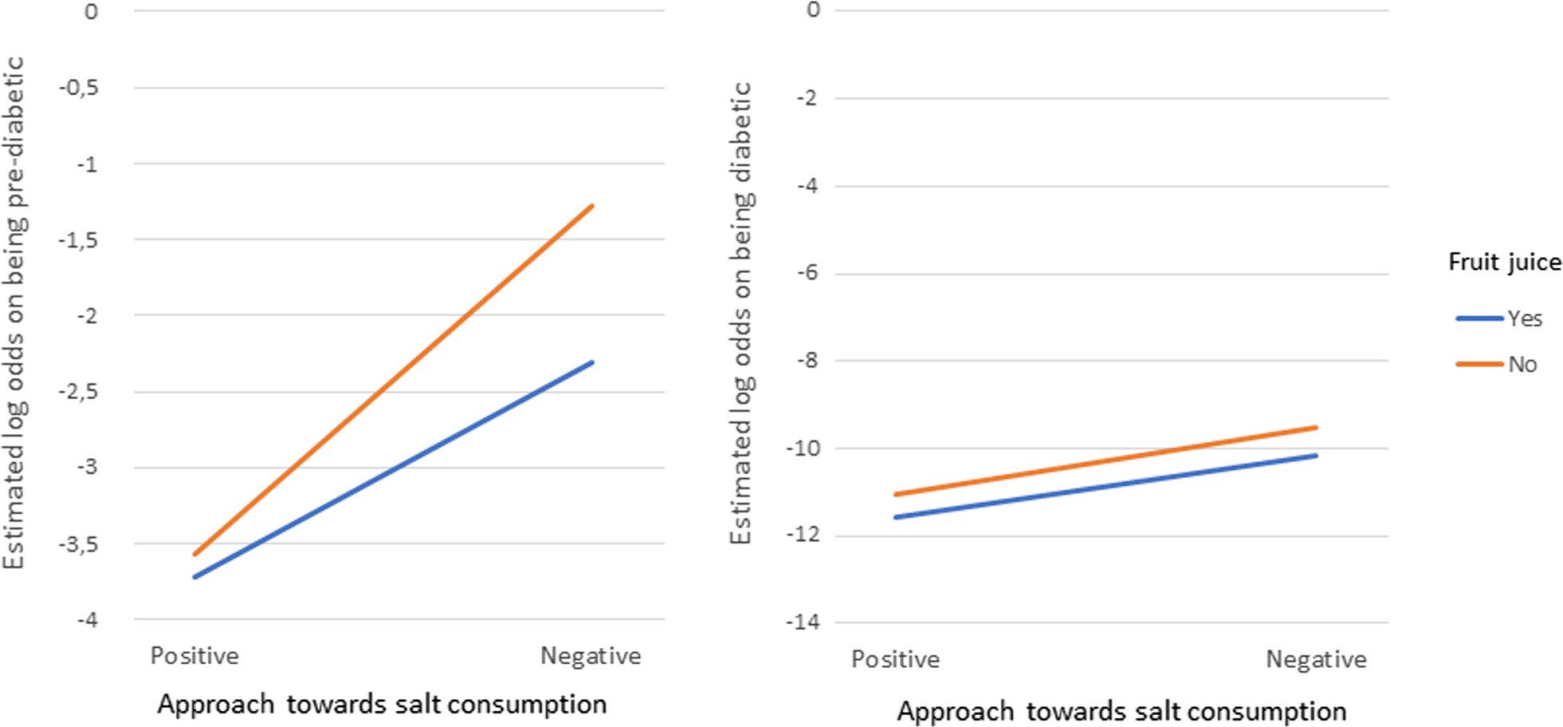
Interaction plot of approach towards salt consumption and consumption of fruit juice

**Fig. 7:**
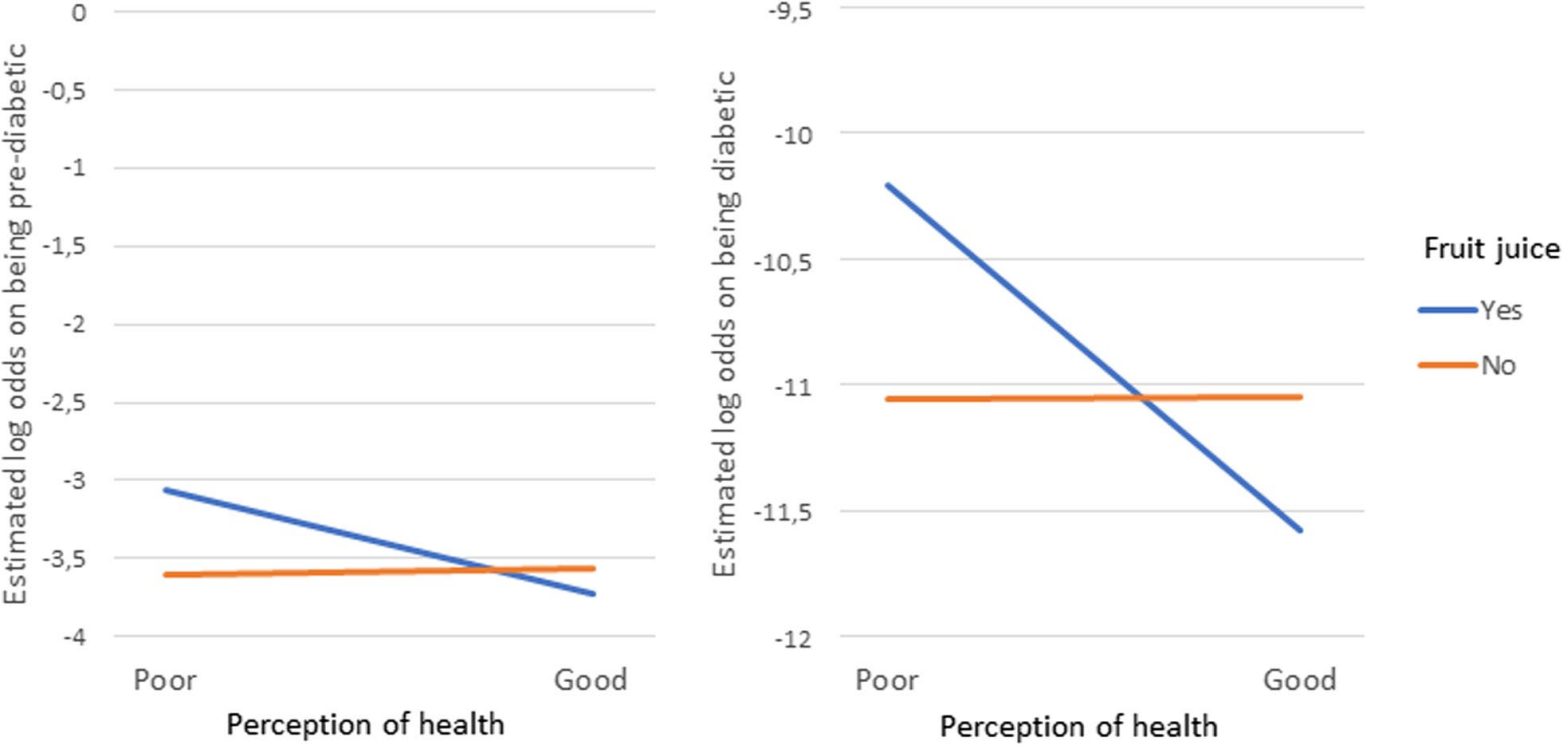
Interaction plot of perception of health and consumption of fruit juice

**Fig. 8:**
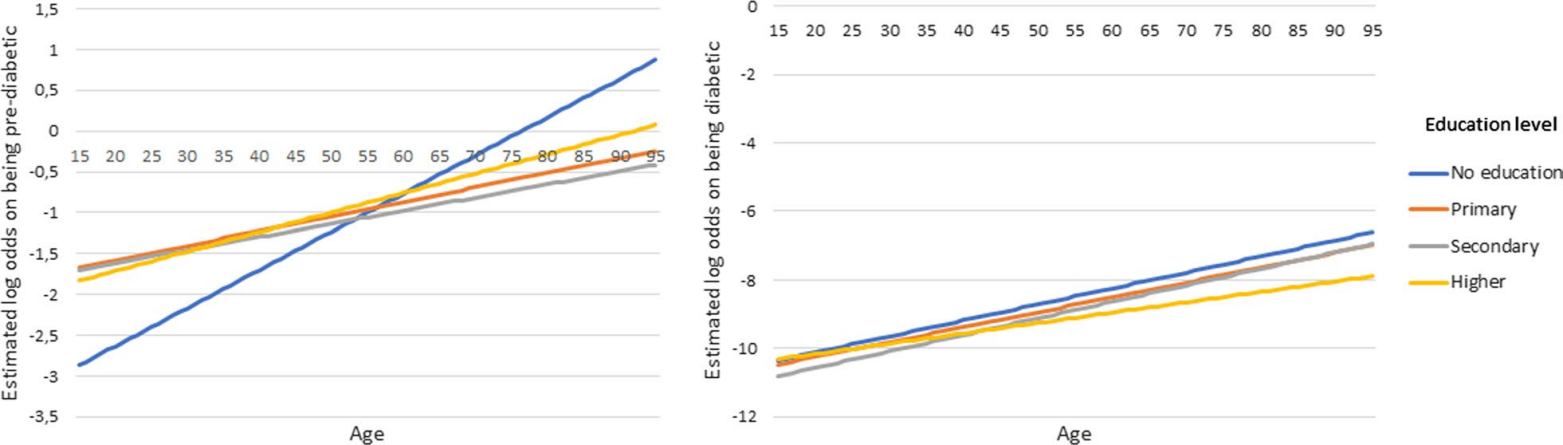
Interaction plot of age and education level

The total effect that the variables included in a two-way interaction effect have on the outcomes are made up of their individual main effects as well as the simultaneous/interaction effect between the two variables. Therefore, the main effects and the interaction effects cannot be interpreted individually. For this reason, the overall effect of these variables involved in the interactions are represented in the form of interaction plots given in Figs. 4, 5, 6 7 and 8. Figures 4, 5, 6 7 and 8 present the estimated log-odds of being pre-diabetic (figures on the left) or diabetic (figures on the right) versus being non-diabetic for each of the interaction effects. A positive log-odds is associated with a higher likelihood of the event, and a negative log-odds is associated with a lower likelihood. It should be noted in Figs. 4, 5, 6 and 7 that for all categories or values of the variables involved in the interaction, there was a lower likelihood of being pre-diabetic and diabetic compared to being non-diabetic (all the estimated log-odds were negative). However, the magnitude of these likelihoods varied according to the categories or values of the variables in the interaction, whereas Fig. 8, which depicts the interaction of an individual’s age with their highest level of education, showed a change from a negative log-odds to a positive log-odds of being pre-diabetic in the older age groups for all education levels. Specifically, individuals aged 65 years or older with no education had the highest log-odds of being pre-diabetic compared to non-diabetic, which can be interpreted as these individuals having the highest likelihood of being pre-diabetic rather than non-diabetic compared to individuals of the same age with any education.

## Discussion

This study aimed to investigate the prevalence and risk factors associated with diabetes and pre-diabetes in the South African population using the nationally representative SADHS 2016 data. This study revealed a substantial proportion of South Africans with pre-diabetes and diabetes who had not been diagnosed prior to the HbA1c test during the survey. Thus, we can infer that a large proportion of the South African population remains undiagnosed.

A generalized additive mixed model was employed in order to account for the design of the study as well as spatial autocorrelation in the data. As anticipated, the model revealed that there were common factors significantly associated with both pre-diabetes and diabetes. These included gender, waist circumference, haemoglobin level (adjusted for altitude and smoking), as well as multiple two-way interaction effects between some of the lifestyle factors, demographic factors and anthropometric measures. However, being diabetic had additional significant factors associated with it. These included blood pressure, taking high blood pressure medication, taking medication, and having smoked within the previous 24 hours of the survey, all of which were not statistically significant with being pre-diabetic. In contrast, Rohrer’s Index was significantly associated with being pre-diabetic but not diabetic. These varying risk factors for pre-diabetes and diabetes confirm the importance of considering diabetic status as a three-level categorical outcome rather than as a binary outcome of simply diabetes versus non-diabetes. Pre-diabetes can be considered as an early indicator for possible progression of T2DM, and thus this condition should be taken seriously. Lifestyle interventions can assist in preventing pre-diabetes from developing into T2DM; however, an individual would be required to be aware of their pre-diabetic status in order to implement these changes. This highlights the importance of studies that contribute to the understanding of the factors associated with pre-diabetes, in addition to diabetes.

The data used in this study revealed that the peak diabetes prevalence is in the 65–100 year old age group. This result is similar to the study done by King et al. [33] who reported that in developed countries, diabetes predominantly occurs in older age groups (65 years and older). The analysis in our study, however, revealed that the effect that age has on the likelihood of pre-diabetes and diabetes is confounded by the individual’s education level, as seen by the interaction between these factors. Particularly, individuals from the age of 65 years old with no education have the highest risk of pre-diabetes. However, this effect of having no education is reversed in the younger age groups, where individuals younger than 53 years old with no education have the lowest risk of pre-diabetes. The interaction effects considered in this study do not only assist in obtaining a better fitting model, but also have important implications concerning the interpretation of the risk factors of pre-diabetes and diabetes. The effect that such factors involved in the interaction terms have on the likelihood of diabetes and pre-diabetes cannot be interpreted independently as it is confounded by other factors. However, many studies ignore such interaction effects.

In this study, participants who were classified as diabetic according to the HbA1c test could not be distinguished between having T1DM or T2DM. However, it is most likely that the majority of these individuals have T2DM as a large percentage of the sample are pre-diabetic and T2DM is the most common type of diabetes [7]. A combination of genetics and environmental factors, such as a diet high in sugar and little physical activity, are contributing factors to T2DM. Measures of central obesity have also been found to be strongly associated with T2DM risk [16, 17, 24]. In fact, T2DM has been found to be so closely related to obesity that the term *diabesity* has been coined [8]. Thus, it is no surprise to see a significant association of the anthropometric measures BMI, Rohrer’s Index and waist circumference with pre-diabetes and diabetes in this study, all of which are indices for obesity. Moreover, our study concurs with the findings by Motala et al. (2008) where it was shown that there exists a positive association between waist circumference and diabetes [17]. In addition, our study found an increased likelihood of pre-diabetes compared to non-diabetes with an increase in Rohrer’s Index and waist circumference.

Our results indicated that individuals who had not smoked in the previous 24 hours were more likely to be diabetic than non-diabetic compared to those that had smoked. This finding contradicts that of Pan et al. (2015), who found that smoking cigarettes was one of the most important modifiable risk factors for diabetes [34]. However, in our study, it must be noted that smoking was based on self-reporting and may not have been accurately reported. Smoking while diabetic is the strongest risk factor for peripheral vascular disease caused by atherosclerosis of the large blood vessels supplying the legs. Thus, the two together can accelerate the progression of peripheral vascular disease and ultimately could lead to the need for amputation [35]. No statistical significance was found in having smoked the previous 24 hours and being pre-diabetic compared to non-diabetic. Individuals taking high blood pressure medication were found to be at an increased risk for diabetes. According to Brunström and Carlberg (2016), such medications should be used with caution as diabetic individuals with a systolic blood pressure less than 140mm Hg that were on anti-hypertensive treatment were at an increased risk of cardiovascular death [36]. No statistical significance was found in individuals taking high blood pressure medication when comparing pre-diabetics to non-diabetics.

Our study also found that individuals taking any medication had a significantly higher risk of being diabetic compared to non-diabetic. Although the type of medication was not disclosed, it is possible that these individuals taking any medication were in poorer health and possibly had more comorbidities compared to those not taking any medication, which may be as a result of health complications caused by diabetes. No statistical significance was found in individuals taking medication when comparing pre-diabetics to non-diabetics.

The limitations of this study include the unavailability of information on some risk factors, such as cholesterol (lipids) level, carbohydrate and fat consumption in the SADHS 2016 data. In addition, the consumption of foods and drink, and having smoked cigarettes, were only recorded for the 24 hours prior to the survey and were based on self-reporting. The data used in this study was based on a cross-sectional survey; therefore, no causal relationship between diabetic status and the factors considered can be established. Future directions of this research include the use of machine learning techniques, such as decision trees, random forests, Bayesian networks and neural networks, for classifying a person’s diabetic status, which can assist general practitioners and healthcare workers as an auxiliary diabetic diagnostic tool.

## Conclusion

This study highlights the high prevalence of pre-diabetes and diabetes in South Africa, as well as the need for individuals to be aware of their diabetic status. Those with pre-diabetes are at a high risk of developing T2DM, especially if they remain undiagnosed. Usually by the time they are diagnosed with T2DM, they have already developed health complications. It is therefore important for all stakeholders in government and the private sector of South Africa to get involved in providing education and creating awareness about diabetes. Regular testing of diabetes, as well as leading a healthy lifestyle, should be encouraged.

## Data Availability

This study utilized existing survey datasets that are in the public domain and freely available from https://www.dhsprogram.com/data/dataset_admin/login_main.cfm with the permission from the DHS Program.

## Abbreviations

95% CI: 95% confidence interval
BMI: Body mass index
GAMM: Generalized additive mixed model
GLMM: Generalized linear mixed model
HbA1c: Glycated haemoglobin
SA: South Africa
SADHS: South African Demographic and Health Survey
T1DM: Type 1 diabetes mellitus
T2DM: Type 2 diabetes mellitus
WtHR: Waist-to-height ratio
